# The molecular mechanisms underlying neutrophil infiltration in vessel co-opting colorectal cancer liver metastases

**DOI:** 10.3389/fonc.2022.1004793

**Published:** 2022-10-18

**Authors:** Miran Rada, Nour Hassan, Anthoula Lazaris, Peter Metrakos

**Affiliations:** Cancer Research Program, Research Institute of the McGill University Health Centre, Montreal, QC, Canada

**Keywords:** crclm, angiogenesis, vessel co-option, neutrophil, Ang1, RUNX1

## Abstract

Colorectal cancer liver metastases (CRCLMs) have two major histopathological growth patterns (HGPs): desmoplastic (DHGP) and replacement (RHGP). The DHGP tumours derive their vasculature by angiogenesis, while the RHGP tumours use vessel co-option. Various studies have associated RHGP tumours with an unfavourable prognosis, as well as high levels of resistance to anti-angiogenic agents and chemotherapy. Recently, we reported higher numbers of neutrophils in the tumour microenvironment (TME) of vessel co-opting tumours compared to their angiogenic counterparts. However, the molecular mechanisms underlying this phenotype are unclear. Herein, we suggested a positive correlation between the expression of angiopoietin-1 (Ang1) in the hepatocytes and the presence of neutrophils in vessel co-opting tumours. Importantly, upregulation of Ang1 in the hepatocytes is associated with the presence of runt-related transcription factor-1 (RUNX1) in the neighboring cancer cells *in vitro* and *in vivo*. Altogether, our data suggest the molecular mechanisms by which neutrophils are infiltrated in vessel co-opting CRCLM lesions. This finding may yield novel therapeutic strategies for CRCLM patients in future.

## Introduction

Colorectal cancer is one of the most common malignant solid tumours globally, and it is characterized by poor prognosis due to high levels of metastases (1). More than 50% of CRC patients generate metastases, and approximately 30% of these patients have liver only metastasis (2). The management of patients with colorectal cancer liver metastases (CRCLMs) is a challenging (3, 4) and only complete surgical resection of liver metastases has the potential to cure (3). However, only 20% of CRCLM patients are eligible for liver resection (5, 6). Therefore, non-resectable CRCLM patients are subjected to a variety of treatment approaches including chemotherapy and anti-angiogenic therapy (7). Unfortunately, limited responses to chemotherapy and anti-angiogenic therapy have been noticed in CRCLM patients (8).

CRCLM lesions have two major histopathological growth patterns (HGPs) including replacement (RHGP) and desmoplastic (DHGP) (7, 9). The cancer cells in RHGP lesions obtain blood their supply by vessel co-option, whereas the DHGP lesions use angiogenesis for their blood supply (9, 10). The cancer cells in vessel co-opting tumours are highly motile, which is mediated by certain proteins involved in the cytoskeleton machinery such as actin-related protein 2/3 (ARP2/3) (7). This feature allows the cancer cells to infiltrate liver tissue and hijack the pre-existing sinusoidal vessels (7, 11, 12). It is worth mentioning that vessel co-option has been reported as a mediator of resistance against anti-angiogenic therapy in CRCLM (7) and other types of cancers including hepatocellular carcinoma, glioblastoma, lung metastases, and melanoma metastases (11, 13–16). 

Exploiting a conditional angiopoietin-1 (Ang1) knockout (Ang1 KO) mouse model, Ibrahim et al. confirmed that Ang1 plays an important role in vessel co-option CRCLM (17). Accordingly, splenic injection of colorectal cancer (MC38) cells into control wild-type mice generated vessel co-opting liver metastatic lesions, while knock out of Ang1 in the liver significantly attenuated the formation of vessel co-opting liver metastatic lesions and induced angiogenic lesions (17). Of note, it has been suggested that Ang1 induces the formation of vessel co-option by promoting cancer cell motility through Tie2-PI3K/AKT-ARP2/3 pathway (18).

Runt-related transcription factor-1 (RUNX1) is a transcription factor that is required for tumour progression and chemoresistance in various cancers (19). RUNX1 is known as a positive regulator of vessel co-option in CRCLM (20). Accordingly, the expression of RUNX1 in the cancer cells promotes cancer cell motility through its target genes including the genes of ARP2/3 complex subunits (20). The upregulation of RUNX1 in the cancer cells is also associated with hepatocyte displacement and replacement by cancer cells in CRCLM (21), which are required for the development of vessel co-option (9).

Neutrophil infiltration has been reported in various types of tumours and they play a crucial role in tumour progression (22). Previously, we demonstrated increased numbers of neutrophil in vessel co-opting CRCLM lesions compared to their angiogenic counterparts (23). However, the molecular mechanisms underlying this phenotype are poorly understood. In this manuscript, we identified the mechanistic pathways by which cancer cells induce neutrophil infiltration in CRCLM. This may lead to novel therapeutic strategies for CRCLM patients with vessel co-option tumours in future.

## Materials and methods

### Cells culturing

The cells (HT29, SW620, IHH and HEK293T) were cultured and maintained in DMEM (Wisent Inc., St-Bruno, QC, Canada, #319-005-CL) supplemented with 10% FBS (Wisent Inc., #085-150) and 1× of streptomycin penicillin/streptomycin (Wisent Inc., 450-201-EL). The cells were incubated at 37°C with 5% CO2.

### Lentiviral shRNA knockdown

We used the following constructs in the current study: Scrambled shRNA#: SHC016, RUNX1 #1: TRCN0000338428; RUNX1 #2: TRCN0000338427 (Sigma Aldrich, Oakville, ON, Canada). We used the calcium phosphate method to transfect HEK293T cells and generate the lentivirus supernatants. The prepared lentivirus supernatant supplemented with 8 µg/mL of polybrene was added to a monolayer of cancer (HT29 and SW620) cells and incubated for 72 h at 37°C with 5% CO2. To select the transfected cells, we treated the cells with 2 µg/mL of Puromycin (Wisent Inc., St-Bruno, QC, Canada, 450-162-XL) for two weeks.

### Immunoblotting

Immunoblotting analysis was performed as described in previous publications (12, 18). Briefly, the lysates were prepared from the harvested cells using RIPA buffer (Sigma Aldrich, Oakville, ON, Canada, #R0278) supplemented with protease inhibitor cocktail (Sigma Aldrich, Oakville, ON #4693124001). The lysates (5μg/sample) were subjected to SDS-PAGE gels and transferred to membranes. The following primary antibodies were used: RUNX1 1:500 (LS Bio, Seattle WA, USA, #LS-C353932), Ang1 1:1000 (Abcam, Waltham, MA, USA, #ab102015), GAPDH 1:2000 (Abcam, Waltham, MA, USA, #ab9485). We analyzed the intensity of the bands with ImageJ software (NIH, Bethesda, MD, USA). All uncropped immunoblotting images can be found in **Supplementary Figure S1**.

### Immunohistochemical staining

We performed immunohistochemical staining for formalin-fixed paraffin-embedded (FFPE) CRCLM sections as described in previous publications (17, 20). Briefly, the sections were deparaffinized, hydrated, and exposed to antigen retrieval and endogenous peroxidase inhibition. The sections were then blocked with 5% goat serum buffer for 1 h at room temperature. The sections were incubated with primary antibodies overnight at 4°C. The sections were washed and exposed to secondary antibodies (Dako, Burlington, ON, Canada, anti-mouse: #K4001; anti-rabbit: #K4003) and incubated for 1 h at room temperature followed by staining with diaminobenzidine (DAB) substrate (Dako, Burlington, ON, Canada, #K3468). The stained sections were scanned and analyzed using (Aperio ScanScope XT System).

We used the following primary antibodies: RUNX1 1:200 (LS Bio, Seattle WA, USA, #LS-C353932), LY6G 1:1000 (Abcam, Waltham, MA, USA, # ab238132) and Ang1 1:1500 (Abcam, Waltham, MA, USA, #ab102015).

### Statistical analysis

We used GraphPad Prism software version 7.0 (GraphPad Software, CA, USA) to analyze the data. The data are presented as mean ± standard deviation. To determine a significant difference between the means of the two groups, we applied unpaired Student’s t-test. We performed the correlation analysis and R-value using Pearson correlation. P-values of <0.05 were considered significant.

## Results

### Knockdown of RUNX1 in the cancer cells attenuates the migration of neutrophils to the tumour microenvironment

The expression of RUNX1 in tumours is positively associated with immune cell infiltration in various cancers including cervical cancer, colorectal cancer, glioma, and renal cancer (24). We previously showed that RUNX1 is upregulated in vessel co-opting CRCLM (12, 20). The vessel co-opting tumours are characterized by high levels of neutrophil infiltration compared to their angiogenic counterparts (23). Therefore, we decided to examine the correlation between RUNX1 expression in the cancer cells and neutrophil infiltrates in CRCLM.

**Figure 1:**
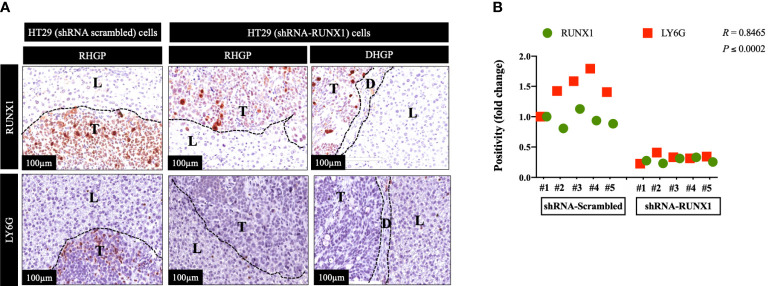
RUNX1 knockdown in the cancer cells attenuates neutrophil infiltration. **(A)** Represents immunohistochemical staining of CRCLM sections generated from HT29 cancer cells expressing shRNA-Scrambled (n=5) or shRNA-RUNX1 (n=5). We used anti-RUNX1 and anti-LY6G (neutrophil biomarker) antibodies. **(B)** Using Pearson correlation analysis, we showed the correlation between RUNX1 expression and LY6G expression in CRCLM sections. Relative expression levels of RUNX1 or LY6G were calculated by dividing the positivity value of each specimen from shRNA-Scrambled and shRNA-RUNX1 groups by the positivity value of specimen #1 from shRNA-Scrambled group and then presenting the result as a fold difference.

We previously generated CRCLM lesions in SCID Beige mice using HT29 cancer cells expressing scrambled or RUNX1 shRNA (20). Accordingly, the presence of shRNA against RUNX1 promoted the formation of angiogenic lesions (20). We used these sections to identify the correlation between RUNX1 expression in the cancer cells and neutrophil infiltration. We performed immunohistochemical staining using RUNX1 and neutrophil marker (LY6G). LY6G is a widely used biomarker of neutrophils in mice (25). As shown in **Figure 1A**, the knockdown of RUNX1 in the cancer cells dramatically attenuated the infiltration of neutrophils into the tumour microenvironment (TME). We found similar levels of neutrophil infiltration in vessel co-opting and angiogenic lesions that were generated by RUNX1-silenced HT29 cancer cells. Importantly, the expression levels of RUNX1 in the cancer cells were significantly correlated with the presence of neutrophils in the TME (**Figure 1B**). Taken together, these data suggest the influence of cancer cells on neutrophil infiltration in CRCLM lesions, and RUNX1 plays a key role in this process.

### The expression levels of RUNX1 in the cancer cells affect the presence of Ang1 in the neighboring liver parenchyma

Ang1 downregulation in the liver parenchyma (hepatocytes) leads to significant decrease of vessel co-option CRCLM in mouse models (17). On the other hand, Burnett et al. (26) have suggested Ang1 as a positive regulator of neutrophil migration *in vitro* and *in vivo*. Accordingly, intraperitoneal injection of matrilin-1-angiopoietin-1 (MAT.Ang-1) significantly enhanced the accumulation of neutrophils compared to saline injection (26). Moreover, they identified Tie2 and CD18 (β2 integrin) as mediators of Ang1-driven neutrophil migration (26).

**Figure 2:**
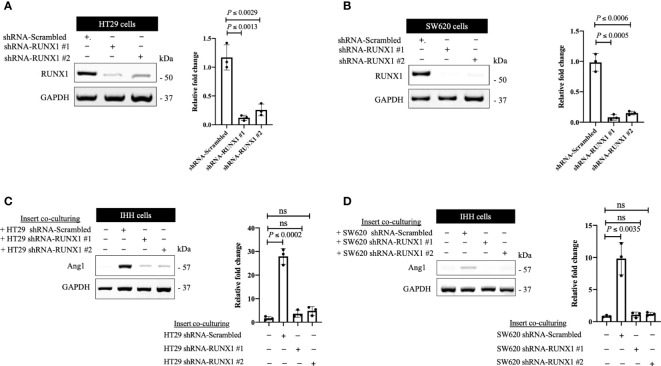
The expression of RUNX1 in the cancer cells influences the expression of Ang1 in adjacent hepatocytes *in vitro*. **(A, B)** Immunoblotting showing the expression of RUNX1 in colorectal cancer (HT29 and SW620) cells (left panels). The right panels represent the intensity of RUNX1 bands after normalization with GAPDH using ImageJ. Data are from three independent experiments. **(C, D)** The left panels show the expression of Ang1 in the co-cultured IHH hepatocytes with colorectal cancer (HT29 and SW620) cells. The right panels show the intensity of Ang1 bands after normalization with GAPDH using ImageJ. Data are from three independent experiments. Data are presented as the mean ± SD. ns, Not significant.

**Figure 3:**
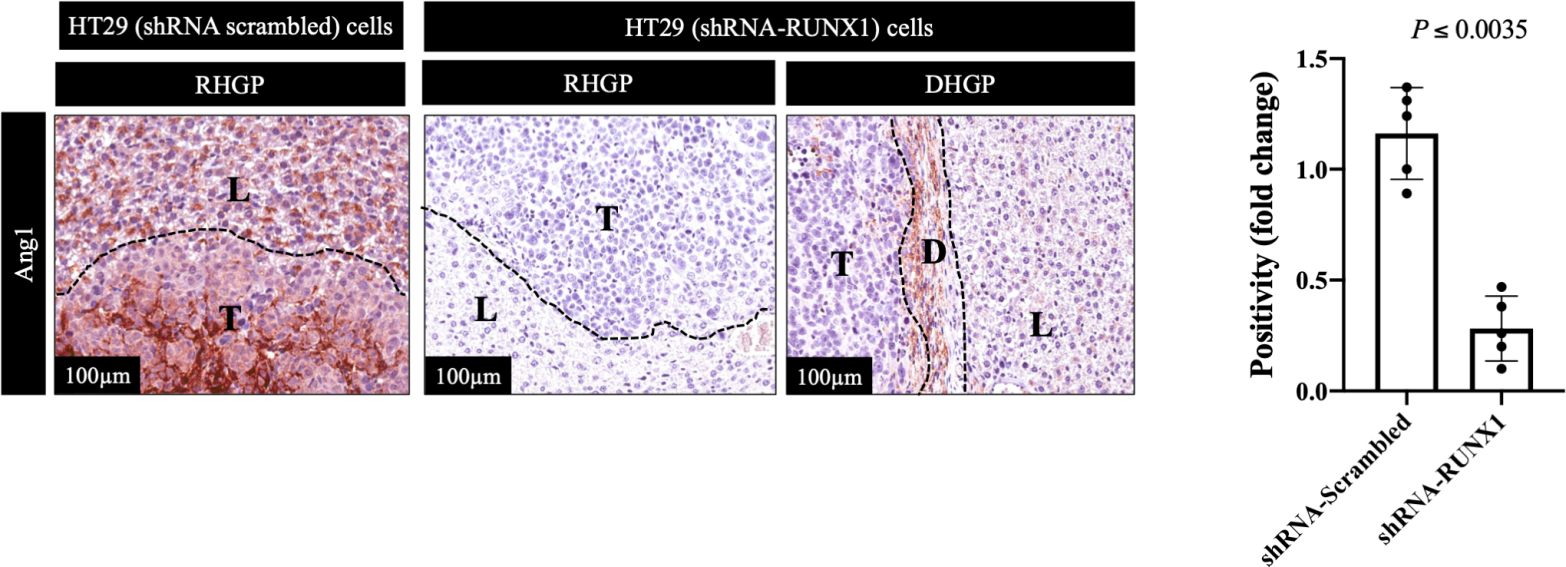
Knockdown of RUNX1 in the cancer cells attenuates the expression of Ang1 in liver parenchyma. The left panel represents immunohistochemical staining CRCLM sections generated from HT29 cancer cells expressing shRNA-Scrambled (n=5) or shRNA-RUNX1 (n=5) using anti-Ang1 antibody. The right panel shows the quantification of total positive staining that was assessed in CRCLM lesions using Aperio software. Relative expression levels of Ang1 were calculated by dividing the positivity value of each specimen from shRNA-Scrambled and shRNA-RUNX1 groups by the positivity value of specimen #1 from shRNA-Scrambled group and then presenting the result as a fold difference.

We previously identified transforming growth factor β1 (TGFβ1) as a positive regulator of Ang1 expression in the hepatocytes *in vitro* and *in vivo* (27). Moreover, we found that RUNX1 positively influences the expression of TGFβ1 in CRCLM through its target genes including THBS1 (20). Therefore, we hypothesised that RUNX1 expression in the cancer cells may be linked to Ang1 expression in the neighbouring hepatocytes. To examine our hypothesis, we decided to co-culture (insert co-culturing) (12, 21) IHH hepatocytes with colorectal cancer (HT29 or SW620) cells expressing either control or RUNX1 shRNA. Firstly, we confirmed the efficiency of RUNX1 knockdown in HT29 and SW620 cancer cells (**Figures 2A, B**). Then we examined the effect of RUNX1 knockdown in the cancer cells on the co-cultured IHH hepatocytes. As shown in **Figures 2C, D**, the expression levels of Ang1 were significantly increased in the hepatocytes upon co-culturing with control cancer cells, whereas silencing RUNX1 in the cancer cells significantly impaired Ang1 expression in the hepatocytes. To further confirm our data, we performed immunohistochemical staining for the same specimens that were used for the experiments in **Figure 1** using anti-Ang1 antibody. These CRCLM specimens were generated previously with HT29 cancer cells expressing control or RUNX1 shRNA (20). Indeed, we observed a significant reduction of Ang1 in the liver parenchyma when the metastases were formed with cancer cells that RUNX1 knocked down (**Figure 3**). We noticed lower levels of Ang1 in CRCLM lesions generated by RUNX1-silenced HT29 cancer cells regardless of their histopathological growth patterns. Collectively, these data suggest RUNX1 as a positive regulator of Ang1expression in the parenchyma of CRCLM lesions.

### The expression levels of Ang1 in the hepatocytes drive neutrophil infiltration into the tumour microenvironment

The overexpression of Ang1 in the host liver has been associated with vessel co-opting CRCLM tumours (17). The function of Ang1 is likely mediated by activation of the Tie2-PI3K/AKT-ARP2/3 pathway, which incites cancer cell motility (18). Notably, high levels of cancer cell motility induce the development of vessel co-opting CRCLM lesions (7).

**Figure 4:**
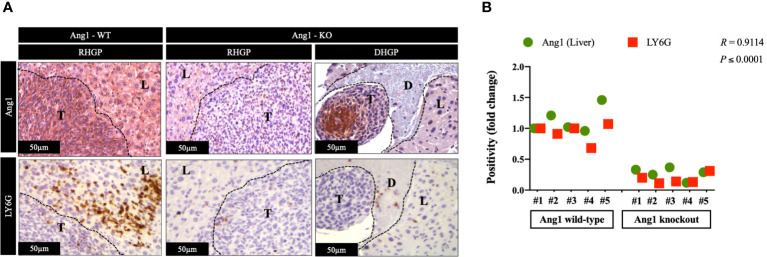
The expression of Ang1 in the host liver positively correlated with neutrophil infiltration. **(A)** Represents immunohistochemical staining of CRCLM sections generated by injecting MC38 cancer cells into wild-type (n=5) and Ang1 knockout (n=5) C57BL/6 mice. We used anti-Ang1 and anti-LY6G (neutrophil biomarker) antibodies. **(B)** Pearson correlation analysis confirmed the correlation between Ang1 expression and LY6G expression in CRCLM sections. Relative expression levels of Ang1 or LY6G were calculated by dividing the positivity value of each specimen from Ang1-wild type (Ang1-WT) and Ang1-kncokout (Ang1-KO) groups by the positivity value of specimen #1 from Ang1-WT group and then presenting the result as a fold difference.

In CRCLM, the vessel co-opting lesions are characterized by higher levels of neutrophil infiltration than their angiogenic counterparts (23). The presence of Ang1 has been correlated with higher levels of neutrophil infiltration *in vivo* (26). Hence, we hypothesized the association between high levels of neutrophil infiltration and upregulation of Ang1 in vessel co-opting CRCLM lesions. We examined our hypothesis by performing immunohistochemical staining for the CRCLM specimens generated from Ang1 wild-type and Ang1 knockout C57BL/6 mice from our previous publication (17). We used anti-Ang1 and anti-LY6G antibodies (**Figure 4A**). Our results suggested a significant correlation between the infiltration levels of neutrophils and Ang1 expression in the host liver (**Figure 4B**). Intriguingly, we notice a comparable effect of Ang1 on the neutrophil infiltration in angiogenic DHGP and vessel co-opting RHGP lesions. Altogether, these data propose that Ang1 upregulation in the host liver favours neutrophil infiltration in CRCLM.

## Discussion

Neutrophils are found to induce tumour progression in various cancers including myxofibrosarcoma, melanoma, pancreatic cancer, hepatocellular carcinoma and gastric cancer (28, 29). Moreover, the elevated numbers of neutrophils in TME has been associated with tumour resistance to chemoradiotherapy (30). Furthermore, we previously reported a positive correlation between neutrophil infiltration and vessel co-option in CRCLM (23), which is responsible for anti-angiogenic resistance in CRCLM (23). However, the molecular mechanisms that underline neutrophil infiltration, as well as the role of neutrophils in vessel co-option are largely unknown. In this manuscript, we mainly focused on the molecular mechanisms of neutrophil infiltration in CRCLM. Our data suggested the RUNX1-Ang1 pathway as a mediator of neutrophil infiltration in CRCLM. However, the impact of neutrophils on the development and protection of vessel co-option has yet to be determined.

It has been reported that tumour-associated neutrophils (TANs) contribute to cancer cell migration, immunosuppression and remodelling of the extracellular matrix (ECM) (31, 32). TANs are known to induce metastases and this function is mediated by releasing inducible nitric oxide synthase (iNOS) (33) and NETs (34); a network structures of extracellular DNA fibres studded with proteins (35). The NET proteins are composed mainly of neutrophil elastase (NE) and matrix metalloproteinase 9 (MMP9) (36, 37). These proteins have been shown to awaken dormant cancer cells mainly through remodeling the extracellular matrix (ECM) (36). We previously reported that the neutrophils in vessel co-opting lesions are characterized by expressing high levels of lysyl oxidase-like 4 (LOXL4) (23). Interestingly, the upregulation of LOXL4 is strongly linked to ECM remodeling and immunosuppressive microenvironment (38). These data suggest that neutrophils are likely involved in ECM remodeling and immunosuppression in vessel co-opting CRCLM lesions. These findings are interesting and further investigation are required to confirm the function of neutrophils in vessel co-option.

The migration of neutrophils toward TME has been investigated extensively (31, 39). Accordingly, the cancer cells play a key role in neutrophil infiltration into TME by producing different signals including chemokines (e.g., IL-8, CCL2, CXCL6) and cytokines (e.g., IL-1β, IL-6, TNFα) (40, 41). Yet, a precise understanding of the factors that contribute to neutrophil trafficking to TME is lacking. Importantly, our data suggested a new mechanism by which cancer cells promote neutrophil migration, which is mediated by the RUNX1-Ang1 pathway. The findings of Burnett et al. (26) supports our work, they found that neutrophils recruitment after injecting mice with recombinant Ang1. Moreover, the expression of Tie2 and CD18 in neutrophils significantly influenced the function of Ang1 (26). The molecules that mediate Ang1-neutrophil interactions in CRCLM are unknown and warrant further investigation.

## Conclusions

**Figure 5:**
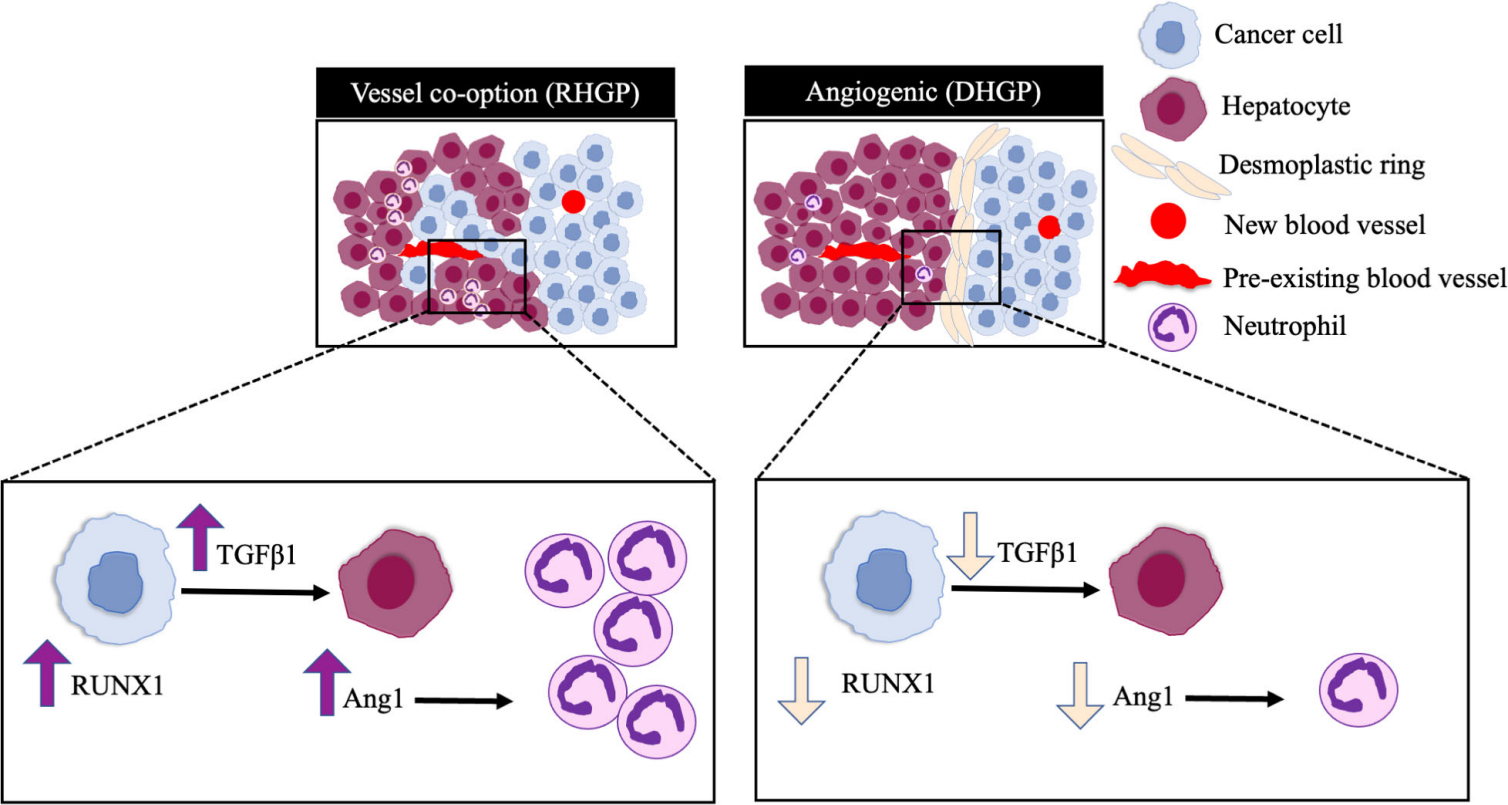
Schematic representation of the pathways involved in neutrophil infiltration in CRCLM. RUNX1 is highly expressed in the cancer cells of vessel co-opting CRCLM lesions compared to angiogenic lesions, which induces the expression of TGFβ1 and Ang1 in the neighbouring liver parenchyma. The overexpression of Ang1 incites the migration of neutrophils into the tumour microenvironment.

This study demonstrated the mechanistic pathways underline high levels of neutrophil infiltration in vessel co-opting CRCLM lesions. Our *in vivo* data revealed RUNX1-Ang1 pathway is responsible for neutrophil infiltration in CRCLM (**Figure 5**). However, the role of neutrophils in the development and maintaining vessel co-option tumours is still an open question and requires further investigation.

## Data availability statement

The original contributions presented in the study are included in the article/**Supplementary Material**. Further inquiries can be directed to the corresponding authors.

## Ethics statement

The animal study was reviewed and approved by McGill University Health Centre Institutional Review Board.

## Author contributions

MR, AL, and PM co-conceived the study. MR executed the experiments. MR performed immunohistochemistry, cell culturing and immunoblotting. NH assisted in immunohistochemistry. MR, data curation, writing and original draft preparation, MR, AL, and PM, review and editing, PM, funding acquisition. All authors have read and agreed to the published version of the manuscript.

## Acknowledgments

The authors would like to acknowledge the support provided by Dana Massaro and Ken Verdoni Liver Metastases Research Fellowship.

## Conflict of interest

The authors declare that the research was conducted in the absence of any commercial or financial relationships that could be construed as a potential conflict of interest.

## Publisher’s note

All claims expressed in this article are solely those of the authors and do not necessarily represent those of their affiliated organizations, or those of the publisher, the editors and the reviewers. Any product that may be evaluated in this article, or claim that may be made by its manufacturer, is not guaranteed or endorsed by the publisher.

## Supplementary material

Supplementary Figure 1 Original uncropped Western blots of **Figure 2**.

Supplementary Table 1 The positivity of the IHC images.

Supplementary Table 2 The intensity ratio of Western blot bands.
