# Supplementary material for: The molecular mechanisms underlying neutrophil infiltration in vessel co-opting colorectal cancer liver metastases
Source: Front Oncol. 2022 Oct 18;12:1004793. doi: 10.3389/fonc.2022.1004793 (PMC9623070; doi:10.3389/fonc.2022.1004793)
Supplement: Supplementary Figure 1 — Original uncropped Western blots of Figure 2 . [file Presentation_1.pptx]

## Slide 1
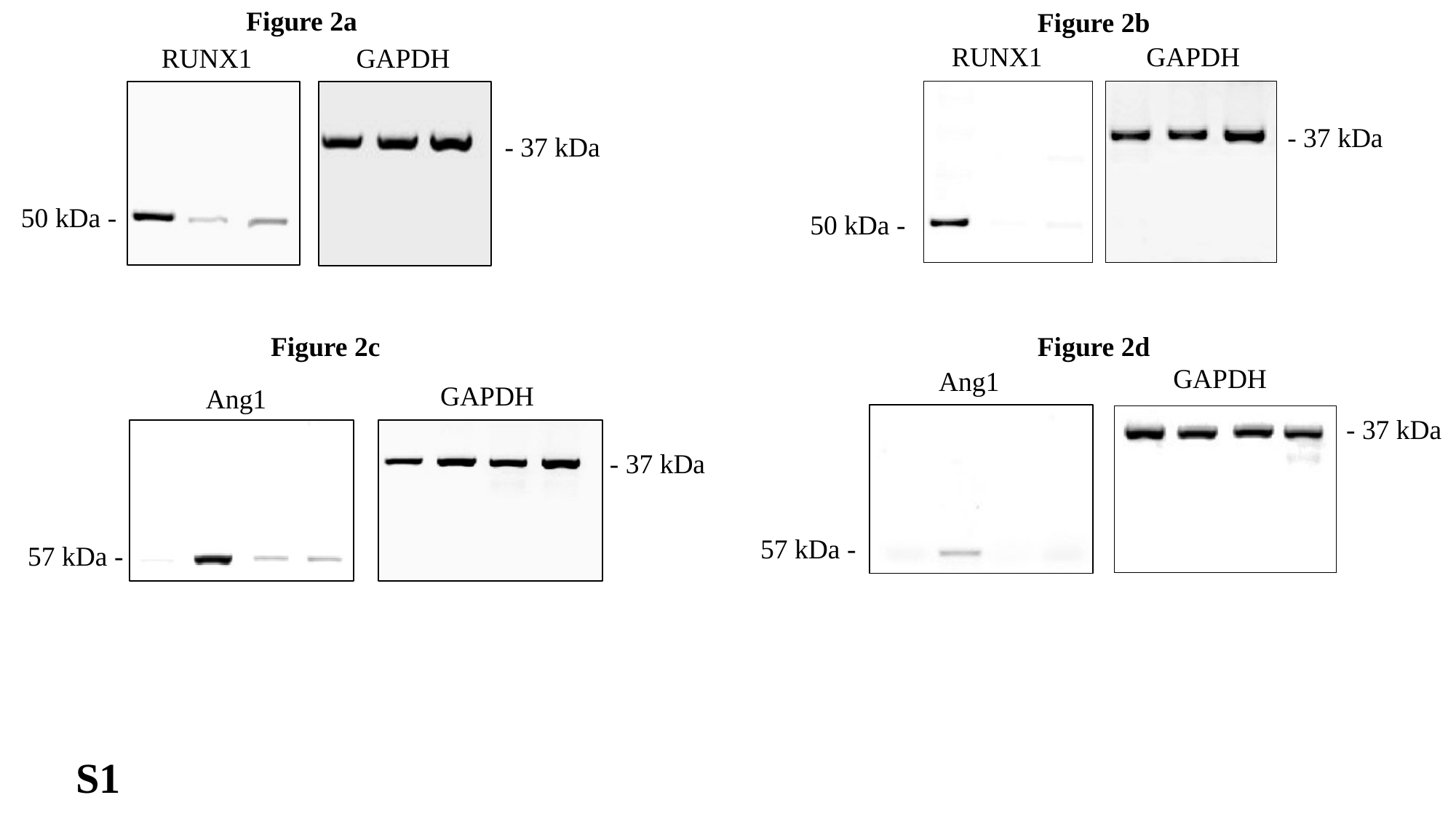

Figure 2b
Figure 2a
RUNX1
GAPDH
RUNX1
GAPDH
- 37 kDa
- 37 kDa
50 kDa -
50 kDa -
Figure 2c
Figure 2d
GAPDH
Ang1
GAPDH
Ang1
- 37 kDa
- 37 kDa
57 kDa -
57 kDa -
S1
